# Individual preferences for physical exercise as secondary prevention for non-specific low back pain: A discrete choice experiment

**DOI:** 10.1371/journal.pone.0187709

**Published:** 2017-12-15

**Authors:** Emmanuel Aboagye, Jan Hagberg, Iben Axén, Lydia Kwak, Malin Lohela-Karlsson, Eva Skillgate, Gunilla Dahlgren, Irene Jensen

**Affiliations:** 1 Institute of Environmental Medicine, Unit of Intervention and Implementation Research for Worker Health, Karolinska Institutet, Stockholm, Sweden; 2 Institute of Environmental Medicine, Unit of Cardiovascular Epidemiology, Karolinska Institutet, Stockholm, Sweden; 3 Department of Public Health and Clinical Medicine, Unit of Occupational and Environmental Medicine, Umeå University, Umeå, Sweden; University of L'Aquila, ITALY

## Abstract

**Background:**

Exercise is effective in improving non-specific low back pain (LBP). Certain components of physical exercise, such as the type, intensity and frequency of exercise, are likely to influence participation among working adults with non-specific LBP, but the value and relative importance of these components remain unknown. The study’s aim was to examine such specific components and their influence on individual preferences for exercise for secondary prevention of non-specific LBP among working adults.

**Methods:**

In a discrete choice experiment, working individuals with non-specific LBP answered a web-based questionnaire. Each respondent was given ten pairs of hypothetical exercise programs and asked to choose one option from each pair. The choices comprised six attributes of exercise (i.e., type of training, design, intensity, frequency, proximity and incentives), each with either three or four levels. A conditional logit regression that reflected the random utility model was used to analyze the responses.

**Results:**

The final study population consisted of 112 participants. The participants’ preferred exercise option was aerobic (i.e., cardiovascular) rather than strength training, group exercise with trainer supervision, rather than individual or unsupervised exercise. They also preferred high intensity exercise performed at least once or twice per week. The most popular types of incentive were exercise during working hours and a wellness allowance rather than coupons for sports goods. The results show that the relative value of some attribute levels differed between young adults (age ≤ 44 years) and older adults (age ≥ 45 years) in terms of the level of trainer supervision required, exercise intensity, travel time to exercise location and financial incentives. For active study participants, exercise frequency (i.e., twice per week, 1.15; CI: 0.25; 2.06) influenced choice of exercise. For individuals with more than one child, travel time (i.e., 20 minutes, -0.55; CI: 0.65; 3.26) was also an influential attribute for choice of exercise, showing that people with children at home preferred to exercise close to home.

**Conclusions:**

This study adds to our knowledge about what types of exercise working adults with back pain are most likely to participate in. The exercise should be a cardiovascular type of training carried out in a group with trainer supervision. It should also be of high intensity and preferably performed twice per week during working hours. Coupons for sports goods do not appear to motivate physical activity among workers with LBP. The findings of the study could have a substantial impact on the planning and development of exercise provision and promotion strategies to improve non-specific LBP. Providers and employers may be able to improve participation in exercise programs for adults with non-specific LBP by focusing on the exercise components which are the most attractive. This in turn would improve satisfaction and adherence to exercise interventions aimed at preventing recurrent non-specific LBP.

## Introduction

Non-specific low back pain (LBP) is a common diagnosis in primary health care affecting 60% to 80% of the population at some point in their lifetime [1]. The condition is characterized by a chain of pain episodes over the life course, i.e. recurrent pain [2]. Aside from the suffering of those affected, LBP constitutes a substantial economic burden in terms of productivity loss in high-income economies [3]. A major part of the rising sickness absence rates, particularly in high-income economies, has been attributed to LBP [4]. The burden of LBP is expected to increase substantially over the coming decades as the population ages [3, 5]. Interventions are thus needed for secondary prevention of non-specific LBP, i.e. prevention of future episodes.

Physical exercise has been shown to be effective for the prevention of recurrent LBP [6]. Since evidence-based medicine became more widely practiced, exercise has been more frequently implemented to prevent future episodes of LBP [7]. This exercise varies from aerobic types and stretching to coordination and strengthening. Some of these interventions have been studied by looking at how individuals value their perceived health benefits. Their cost-effectiveness has been evaluated in health outcome research [8, 9].

The components of exercise interventions vary widely. There is no evidence that one particular exercise intervention is clearly more effective than any other [10, 11], which implies that more evidence is needed to improve our understanding of what really works for each individual. In using evidence-based recommendations of exercise, differences between individuals can be a problem which results in poor adherence to exercise recommendations [12]. There have been various recommendations to enhance the care given to individuals and improve adherence, mainly in the form of trainer supervision. Another suggestion from evidence-based practice is the importance of taking the individual’s values and preferences into decision when choosing exercise interventions [13].

A number of studies have suggested that exercise interventions that are individually designed, instructor-led and high-intensity are associated with improved adherence rates [11, 12]. Structured exercise interventions that are incentivized (i.e. by offering certain benefits) can increase participation rates [14, 15]. There is, however, limited evidence regarding the relevance of employer-incentivized exercise interventions for working adults with non-specific LBP. The employer perspective is relevant in that the incentive component can be used to motivate working adults who otherwise would choose not to exercise because it is too expensive or would not prioritize it.

Apart from these exercise characteristics, individual characteristics (such as background factors, lifestyle and attitude) have been found to be associated with adherence rates for physical activity recommendations [16]. This implies that individual differences in health choices and adherence are associated with individual attitudes towards and motivation to comply with exercise recommendations. Furthermore, factors such as social support, stages of change, general physical health and prior adherence to physical activity have been shown to be determinants of engagement in physical activity [17, 18]. Observational studies conducted to gain insight into the potential environmental determinants of physical activity among adults identified that having a companion to exercise with, the availability of equipment and effective travel channels to engage in physical activity are positively associated with a number of types of physical activity [17]. Thus individual factors, environmental factors and rewards could inform patterns of response to and choice (i.e. preferences) of exercise when individuals are presented with two or more alternatives. However, there is generally little research into individual exercise preferences. Specifically, we need to look at which components of exercise are most attractive.

Understanding individual preferences is important since some studies have shown improvement in important clinical outcomes, such as reduced pain and improved back function, when individuals have been offered their preferred treatment option [19]. Although individual preferences may not in themselves yield the intended health benefits, certain individual-related and exercise characteristics can impact on preference and adherence rates that in turn affect outcomes. Given the many factors that determine what form of exercise is best suited to an individual, taking individual preferences into consideration may be important when recommending exercise interventions, particularly for individuals with strong preferences [19, 20]. The unique contribution of this study is that it integrates the various determinants of physical activity, including, environmental factors (e.g., social support and proximity), individual factors (attitudes) and worksite organizational factors (incentives) to examine preferences from among a wide variety of components of exercise. A stated preference method can be used to understand which components of exercise are valued most by individuals, the relative importance of the components in individual preferences and the role of employer incentives with regard to exercise choices.

The aim of this experiment was therefore to examine specific components of physical exercise and their influence on individual preferences. The relative importance of these components in the choice of exercise for secondary prevention of non-specific LBP among working adults was also investigated.

## Materials and methods

The study used a Discrete Choice Experiment (DCE), which is an attribute-based measure of utility derived from consumption of goods or services. The experiment is based on the assumption that goods and services, in this case exercise, can be described by their components (e.g. training type, design, intensity and frequency). The individual’s values and preferences for exercise depend on these components (also defined as attributes and levels) [21, 22]. In other words, the choice of exercise for secondary prevention of non-specific LBP is influenced by individual preferences for a certain type of training or intensity. A DCE was suitable for the aim of this study because the contribution of each component of a physical exercise option that led to a participant’s choice could be efficiently estimated [23, 24].

### Sampling and data collection

This study recruited participants through a network of occupational health services (OHS) and primary care clinics in Sweden. All the study participants were consecutively recruited by clinicians (i.e., physiotherapists, chiropractors and naprapaths) who manage LBP.

The inclusion criteria were working adults aged 18 ‒ 65 who consulted for non-specific LBP and at the time had not been sick-listed for more than 14 days. The primary pain site for inclusion was pain in the lower back with or without secondary pain sites (pain in other parts of the body). Individuals with specific back pain, prior spine surgery, severe comorbidities such as chronic heart failure, tumors causing physical disability and serious mental illness (eg. depressive disorders) that could affect the ability to perform exercises were excluded, as were those whose command of Swedish was insufficient. The clinicians recruiting subjects for the study obtained written informed consent and forwarded the individual’s contact details to the research centre. The contact details were used for correspondence with study participants and to send an email with a link to the web-survey.

In DCEs, the appropriate sample size selection depends on factors such as the choice tasks, number of attributes, availability of respondents, heterogeneity of target population and the need to conduct subgroup analysis [25]. Traditionally, the rule of thumb for estimating sample size in DCEs is given as: n x t x a/c ≥ 500 [26]. Where n = number of expected respondents, t = number of tasks (10), a = number of alternatives per task (2), c = maximum number of levels (4). In this study, n ≥ 500 x 4/ (2 x 10) = 100. Therefore, the sample size (n) should be ≥ 100.

It has however been suggested that the appropriate sample size should be increased if the study aims to perform subgroup analyses [27]. Our initial target was therefore to recruit 300 subjects in order to allow for subgroup analysis. Thus any additional sample above the 100 would improve the efficiency of the estimated effect parameters. This follows good practice with regards to choice of sample size in DCEs in health care settings [28].

#### Web-based survey

A Swedish-language web survey was administered from March 2015 to March 2016. Reminders were sent out twice at one-week intervals to non-respondents.

The survey consisted of two parts. The first, general part, contained questions related to the individual’s background characteristics, their job demands, general health status, neck and back pain and stages of change in relation to physical activity. Study participants were asked about job demands (i.e. physical, psychological or both). A general health questionnaire was used to assess health status [29]. Each respondent’s stage of change, readiness to exercise and attitudes towards physical exercise were also assessed [30]. Neck and back pain were assessed using parts of a questionnaire for classifying pain status [31]. Bothersome pain [32] was assessed by the question: How many days during the past week has your low back pain been bothersome, i.e. affected your daily activities or routines? Reponses were scored from 0 to 7.

#### Attributes and levels

The second part of the web-survey was the choice experiment. The attributes and levels of the choice experiment were formulated after performing a literature search and a series of group discussions with physiotherapists, chiropractors and research experts on physical exercise. A number of expert meetings were held to discuss which components of physical exercise should be included in the choice experiment design.

The criteria for the initial selection of attributes and corresponding levels were based on components of physical exercise that have previously been shown to improve adherence to physical activity among adults with non-specific LBP [12]. The attributes associated with factors that enhance physical exercise participation are type of training, design, intensity, frequency of exercise. Proximity to the location (e.g. gym space), which is likely to affect regular participation in the exercise, was also included, with three levels for proximity ranging from 10–30 minutes. Financial incentives generally have a positive effect on an adult health-related behaviors and choices [14, 33]. An additional four levels of financial incentive were therefore selected, based on the notion that employers can choose to support physical exercise among working adults. The incentives included were: allowing training during working hours; giving discount coupons for sportswear or fitness; wellness subsidies (i.e. contributing to fees); and no incentive.

The attributes and levels that were arrived at by this process were used to design a choice experiment. After that, a rigorous pilot test on a sample (n = 20) of relevant respondents (i.e. working adults consulting OHS or primary care clinics for LBP) was conducted to check for problems in interpretation and face validity. The group of subjects who were to determine whether the questions could be improved and made applicable to the population of interest was selected based on the same inclusion criteria. They were all recruited from two chiropractic clinics, mostly female with children, age 40 years on average and generally with low back or neck pain when they answered the questions. In the pilot test, participants were to determine whether they understood the definitions of the attributes and levels. Open-ended responses showed that respondents understood the task.

The six attributes in Table 1 were used, in the main survey, for the final design of the choice experiment after responses from the pilot study had confirmed the suitability of the selected attributes and levels.

**Table 1:** Description of physical exercise by its components.

Attributes	Attribute levels	Description
Type of training	Strength training	The kind of exercise that is performed (running, stretching, yoga etc.).
	Cardiovascular training	
	Mindfulness-based training	
Design	Individual with supervision	Decision to exercise individually or in groups, and exercise that is supervised or not supervised.
	Individual without supervision	
	Group with supervision	
	Group without supervision	
Intensity	Low	The degree of effort required to perform the physical exercise.
	Medium	
	High	
Frequency	Once a week	The number of times per week the exercise is performed.
	Two times per week	
	Three times per week	
Proximity	10 minutes	Travel time to the location (e.g. gym) where you can exercise regularly.
	20 minutes	
	30 minutes	
Incentives	None	Describe incentives to receive from an employer as motivation for exercising
	Discount coupon for sports goods	
	Wellness subsidies	
	Exercise in working hours (1h per week)	

### Experiment design

The survey contained the six attributes of physical exercise, each with three or four levels. A large number of unique physical exercise options (1296 choice tasks) can be created from this number of attributes and levels. For practical purposes it was not possible to present every possible combination of attribute and level to the respondents. For this reason, a random and statistically efficient non-orthogonal design, based on usual design principles and practice in DCEs [21, 34], ensured that each level had the likelihood of appearing approximately the same number of times with minimal level overlap. The choice tasks were randomly created for main effects estimation from the attribute using the conjoint survey design tool [34]. This resulted in ten pairwise comparisons of exercise options. Each choice task consisted of two alternative hypothetical exercise options. Each respondent was shown ten pairs of physical exercise options and asked to choose the option they preferred from each pair (Table 2). See also S1 File for the choice task questions.

**Table 2:** An example of a choice task.

Attributes	Alternative A	Alternative B
Type of training	Strength training	Strength training
Design	Individual without supervision	Individual without supervision
Intensity	Medium	Medium
Frequency	Two times per week	Once a week
Proximity	10 minutes	10 minutes
Incentives	Exercise in working hours	Wellness subsidies
**Which exercise option do you prefer?**	[ ]	[ ]

The respondents were not given an opt-out option, given that physical activity is recommended for individuals with non-specific LBP, and we were interested in establishing the exercise attribute preferences among both highly-motivated and non-motivated individuals.

### Statistical analysis

The analysis of DCE data is based on the random utility model, in which the utility associated with an alternative becomes a function of the observed characteristics (i.e. attribute levels) and the unobserved characteristics of the alternative. Thus the observed characteristics influencing participants’ utility of choosing exercise option A or B can be specified as:

$$U_{i}=V\left(\beta ,\;X_{i}\right)+\varepsilon_{i}$$

where V is a function of the attribute levels and reflects the utility (U) derived from the choice of a physical exercise, ε refers to the error term (i.e. unobserved characteristics), X_*i*_ is a vector of attribute levels defining alternative i, and β is a vector of the estimated coefficients.

The key assumption of the model is that each respondent will choose the alternative that provides the highest utility for all alternatives for each choice task. The error term in the model may be due to unobserved attributes affecting choice, differences at individual level owing to heterogeneity in preferences, and any measurement errors or functional specification. All the attribute levels are dummy-coded in the analyses. Consequently, each p-value is a measure of the statistical significance of the difference between the estimated preference weight and the omitted category [35], i.e. the reference category.

In previous studies, choice data from a two alternative forced-choice DCE have often been analyzed using the probit or logit regression model [36]. In the present study we analyzed data using a conditional logit to estimate the probability of choice between two alternatives. A conditional logit was used because it has been shown to be consistent with the random utility model of choice [37].

Multinomial logit that has similar statistical assumptions to conditional logit was also used to model the relationship between choices and the background characteristics of the respondents [37]. Covariates, including age (AGE), sex (SEX), educational level (EDU), annual income (INC), number of children at home (NCHL), job demands (JOB), physical activity level (PHY) and bothersome pain (BOTHER) were included to test their influences on choice of an exercise option such that:

$$Exercise\;choice=\beta_{0}+\beta_{1}AGE+\beta_{2}SEX+\beta_{3}EDU+\beta_{4}INC+\beta_{5}NCHL+\beta_{6}JOB+\beta_{7}PHY+\beta_{8}BOTHER+\varepsilon$$

We tested whether preferences for component of exercise varied by respondents’ background characteristics. The variable age was dichotomized with equal width interval of two groups above and below the mean age. Physical activity level was categorized according to frequency of leisure time exercise. Inactive individuals are those who reported they did no exercise at all as well as those who reported exercising only once a week because of the tendency of individuals to over report exercise behavior [38]. Study participants were also categorized according to those with no children and those with at least one child at home.

STATA software version 12 was used to estimate preference weights (i.e. the Beta coefficients) and standard errors for the conditional and multinomial logit models. Statistically significant (p < 0.05) coefficients indicate the importance of that attribute level in maximizing utility for the stated preference. Internal validity was also determined by checking whether the coefficients (attribute parameters) moved in the expected directions.

Approval to conduct the study was granted by the Regional Ethics Review Board in Stockholm with diary number 2014/2004-31/4.

## Results

A total of 173 working adults with LBP from clinics all over Sweden were invited to participate in the survey. Six individuals were excluded from the study for not fulfilling the inclusion criteria while nine declined to participate. Another 45 of 158 subjects who received the survey did not respond, which gives a response rate of 113 participants or 72%. One participant was excluded for not completing the survey. Participants were not excluded if internal background data was missing (n = 11). As a result, 112 participants were included in the analysis. Each participant provided responses to ten completed choices, resulting in 2240 observations (i.e. 112 participants x 10 choices x 2 options for each choice).

The summary of background characteristics for the study sample is presented in Table 3. Since participants received the survey about a month on average after they were recruited, some individuals were without back pain at the time of the survey but were included in the analysis because they suffered from recurrent back conditions, with most subjects experiencing pain-free periods. The mean number of days of bothersome pain per week was 2.8. According to the frequency of exercise in leisure time, around half of the sample reported that they were physically active. Respondents had an average annual gross income of SEK 250000 (SEK 9.6 = $ 1 in 2016) or higher.

**Table 3:** Background characteristics of the study sample (n = 113).

Category	Characteristics	Number of participants (SD or %)
Age (n = 112)	Mean age (SD)	44 (10.4)
	≤ 44 years	58 (52%)
	45+ years	54 (48%)
Sex (n = 113)	Female	68 (60%)
	Male	45 (40%)
Education level (n = 112)	Basic/ secondary	64 (57%)
	Higher education	48 (43%)
Annual gross income (n = 111)	≤ than SEK 200000	7 (6%)
	SEK 200000–399999	58 (52%)
	SEK 400000–599999	30 (27%)
	≥ than SEK 600000	16 (14%)
Number of children at home (n = 106)	Median (range)	1 (0–9)
	No children	48 (45%)
	1 or more children	58 (55%)
Job demands (n = 111)	Psychologically demanding	54 (49%)
	Physically demanding	17 (15%)
	Physically & psychologically demanding	40 (36%)
Exercise in leisure time (n = 111)	No exercise at all	7 (6%)
	Exercise sometimes	19 (17%)
	Exercise regularly about once a week	11 (10%)
	Exercise regularly about twice a week	37 (33%)
	Exercise at least twice a week	37 (33%)
Physical activity level (n = 111)	Active	74 (67%)
	Inactive	37 (33%)
Neck/ back pain currently (n = 112)	Yes	83 (74%)
	No	29 (26%)
No. of bothersome pain days/week (n = 111)	Mean number of days 0–7 (SD)	2.8 (2.7)
	0	35 (32%)
	1	10 (9%)
	2	13 (12%)
	3	16 (14%)
	4	6 (5%)
	5	7 (6%)
	6	1 (0.9%)
	7	23 (21%)
General health status (n = 112)	Very Good	13 (12%)
	Good	63 (56%)
	Fair	29 (26%)
	Bad	7 (6%)
	Very bad	0 (0%)

SEK = Swedish Crowns (SEK 9.6 = $ 1 in 2016). Physical activity level is categorized according to frequency of leisure-time exercise. Inactive ranges from no exercise at all to exercise regularly about once a week.

The results of the decision analysis are presented in Table 4. The attribute levels with higher positive coefficients (preference weights) are preferred to those with lower weights. Attributes that are significant have an impact on the probability of choosing an alternative.

When all else has been taken into consideration, the most popular exercise option was high intensity cardiovascular training two times a week in a group with trainer supervision. Travel time (i.e. proximity) was not of importance for choice of exercise. Exercising during working hours was the most preferred type of incentive. An hour of exercise during working hours was marginally preferred to the wellness subsidy. Both were preferred over no incentive at all, while discount coupons for sports goods were least popular.

**Table 4:** Relative preference weight and the importance of the attributes for choice (n = 112).

Attribute levels	Coefficients	p-value	95% CI
Cardiovascular training	1.10	**<0.001**	0.75; 1.46
Mindfulness-based training	0.18	0.262	-0.13; 0.49
Strength training	Set to 0		
Individual with supervision	0.45	0.136	-0.14; 1.05
Individual without supervision	0.92	0.093	-0.15; 1.99
Group with supervision	1.29	**<0.001**	0.75; 1.83
Group without supervision	Set to 0		
Low intensity	0.29	0.525	-0.61; 1.19
High intensity	1.51	**<0.001**	0.75; 2.27
Medium intensity	Set to 0		
Once a week (Frequency)	0.78	**<0.001**	0.42; 1.14
2 times/ week (Frequency)	1.66	**<0.001**	1.27; 2.06
3 times/ week	Set to 0		
Proximity (10 minutes)	0.34	0.407	-0.47; 1.15
Proximity (20 mintues)	0.53	0.254	-0.38; 1.44
Proximity (30 mintues)	Set to 0		
None (Incentives)	1.95	**<0.001**	1.07; 2.82
Wellness subsidies	2.71	**<0.001**	1.74; 3.69
Exercise at work	2.78	**<0.001**	1.87; 3.68
Discount coupon for sports goods	Set to 0		

Dependent variable: Choice of exercise option A or B; Set to 0 (Zero) = Reference category (different levels were used as the reference category for easy interpretation of coefficients)

Number of observations = 2240; Log likelihood = -1213.75; CI: Confidence interval

The relative importance of the attributes for exercise preference is given by the difference between the highest and lowest attribute level parameters, divided by the sum of the differences across all attributes. The relative importance value in percentages reveals how respondents valued specific exercise component in relation to the other studied components. The relative importance of ‘exercise intensity’ was on average 25%; ‘type of exercise’ 19%; ‘frequency’ 18%; ‘exercise and supervision levels’ 17%; and ‘incentive’ 17%. The relative importance of ‘proximity to place of exercise’ was on average 4%. Thus the intensity of the exercise was the most important attribute. Travel time to the exercise location was subordinate to all other attributes.

### Background factors associated with choice of physical exercise

The background characteristics that influence choice of exercise and the values given to exercise attributes were examined to determine whether preferences varied systematically between respondents. The analysis reveals a statistically significant relationship between age and exercise preference. Subgroup analysis of how preferences differed between age groups is presented in Table 5. See also S1 Tables for complementary sub-group analyses. The results show that the relative value of some attribute levels differed between young adults (Age ≤ 44 years) and older adults (Age ≥ 45 years) in terms of the level of trainer supervision required, exercise intensity, travel time to exercise location and financial incentives. For active study participants, exercise frequency (i.e. twice per week, 1.15; CI: 0.25; 2.06) influenced choice of exercise. For individuals with more than one child, travel time (i.e. 20 minutes, -0.55; CI: 0.65; 3.26) was also an influential attribute for choice of exercise, showing that people with children at home preferred to exercise close to home.

**Table 5:** Sub-group analysis of exercise preferences influenced by individual characteristics, age group.

Attribute levels	Coefficients [Age ≤ 44], n = 58	Coefficients [Age ≥ 45], n = 54	Difference [Coefficients]	p-value	95% CI
Cardiovascular training	1.49	0.79	0.71	0.055	-0.02; 1.44
Mindfulness-based training	0.19	0.16	0.032	0.922	-0.61; 0.67
Strength training	Set to 0	Set to 0	-		
Individual with supervision	-0.08	0.88	-0.96	0.126	-2.19; 0.27
Individual without supervision	2.79	-0.82	3.62	**<0.001**	1.42; 5.81
Group with supervision	1.59	1.09	0.50	0.364	-0.59; 1.59
Group without supervision	Set to 0	Set to 0	-		
Low intensity	1.87	-1.13	3.00	**<0.001**	1.16; 4.84
High intensity	2.89	0.32	2.58	**<0.001**	1.02; 4.15
Medium intensity	Set to 0	Set to 0	-		
Once a week (Frequency)	0.67	.97	-0.30	0.412	-1.03; 0.42
2 times/ week (Frequency)	1.62	1.86	-0.24	0.559	-1.05; 0.57
3 times/ week	Set to 0	Set to 0	-		
Proximity (10 minutes)	-0.92	1.57	-2.49	**0.003**	-4.14; 0.83
Proximity (20 mintues)	-0.99	1.96	-2.95	**<0.001**	-4.81; 1.09
Proximity (30 mintues)	Set to 0	Set to 0	-		
None (Incentives)	3.17	0.95	2.22	**0.015**	0.43; 4.02
Wellness subsidies	4.05	1.67	2.38	**0.020**	0.37; 4.39
Exercise at work	4.08	1.75	2.33	**0.014**	0.47; 4.19
Discount coupon for sports goods	Set to 0	Set to 0	-		

Number of observations (Age ≤ 44) = 1160; Number of observations (Age ≥ 45) = 1080; Set to 0 (Zero) = Reference category

In general, the coefficients (attribute parameters) that were statistically significant moved in the expected directions and demonstrated the level of importance in influencing choice of physical exercise: see Tables 4 and 5.

## Discussion

The effectiveness of exercise for secondary prevention of recurring non-specific LBP is well established, at least for those who adhere to the training. The current evidence shows that a number of exercise interventions have similar efficacy, but their components vary widely [10, 11]. Little is known about how individuals value specific components of physical exercise, such as type, intensity or frequency. Thus, it may be difficult for clinicians and employers to know which available options to implement and recommend in order to maximize adherence and outcome. This study aimed to examine specific components of physical exercise and their influence on exercise preferences in order to improve non-specific LBP among working adults.

The study findings suggest that the preferred exercise among working adults with non-specific LBP was cardiovascular training of high intensity, performed in a group with trainer supervision once or twice per week. A non-financial employer incentive (i.e. exercise during working hours) was the most preferred option, closely followed by a wellness subsidy. Discount vouchers for sports goods were least popular. The results demonstrate that there is a potential for the use of employer-paid incentives to improve physical activity among working adults with non-specific LBP. Employers should know that employees prefer exercise incentives in the form of breaks from work to exercise or subsidies for the time used to exercise.

Previous research has looked at the role of incentives in motivating physical exercise, with findings suggesting that the use of rewards by employers could encourage healthy behaviors. The results of this current study corroborate the conclusions from previous research regarding incentives used to motivate physical activity [14, 33, 39]. The use of vouchers for sports goods should not be recommended to motivate physical activity among workers with LBP.

Exercise intensity was the most important attribute for exercise choice. Most of the participants (67%) considered themselves to be physically active prior to the study. One can therefore assume that these study participants were not ignorant of the benefits of physical activity and that they associated high intensity exercise with ‘better outcomes’ for non-specific LBP. Physical exercise is a preventive factor for recurrent LBP [6]. Previous research has also shown that excessive physical exercise and inactivity are both associated with increased risk for back pain. However, physical exercises excluding sporting activities have not been found to be associated with LBP when examined by the intensity of physical exercise [40].

Travel time (i.e. ‘proximity to the location of exercise’) was less influential than other attributes with regard to preferences for exercise among individuals with non-specific LBP. This could be explained as an inability of the model used to estimate the coefficient efficiently, or as heterogeneity in the preference for this attribute. The subgroup analysis showed that the older adults (i.e., > 45 years) preferred longer travel time to shorter travel time when choosing exercise. This appears to be counterintuitive, since one might assume that older people would be averse to traveling long distances [41]. Franco, Howard [41], however, had a much older subgroup sample than those in this current study with a gainfully-employed working population. Our findings may reflect the fact that older people do not have young children at home and are thus not as time sensitive as people with young families. This is supported by our results which indicate that number of children affects the importance of travel time.

It is clear that young workers were more inclined than their older colleagues to be motivated by financial incentives when choosing exercise. No employer incentive was associated with a higher probability of choosing exercise than if coupons for sports goods were offered. The nature of the incentives on offer could help to explain this result. The voucher incentive was less attractive than those that improved employees’ chances of physical activity. This implies that the type of incentive may matter when trying to encourage the uptake of physical activity [42]. It could also be that this largely physically active sample responds little or not at all to less attractive incentives [43] or that the incentives undermine their intrinsic motivation [44]. This implies that financial incentives may matter, but consideration should be given to what trade-offs individuals face to improve participation in physical activity.

When looking at respondents’ background characteristics as an effect measure modification of exercise preferences, the results showed that exercise preference differed between age groups with regard to level of trainer supervision, intensity, distance and financial incentives. For instance, study participants who saw themselves as active chose a higher frequency than the non-active group. This implies that individual-related factors can influence choice, and perhaps acceptance and compliance rates. Decision makers (i.e. clinicians and employers) should combine the various the determinants of physical activity to find ways to engage people in physical activity. There were no clear differences in preference according to sex, educational attainment, income, job demands, physical activity level or bothersome pain.

One of the strengths of this study design is that the relative preference information that the DCE provides is immediately understood and helpful for policy implementation. If we assume that the exercise design or financial incentives influence adherence, and that adherence in turn influences health outcomes, the specific components of exercise interventions that influence preferences should be taken into consideration by providers and employers when recommending exercise for working adults. This would enhance individual satisfaction and thus adherence to exercise interventions aimed at secondary prevention of non-specific LBP.

This study has some limitations. There were two phases followed to arrive at the attributes and levels used in this experimental design. Firstly, a literature search and a series of expert group discussions were conducted. Secondly, a pilot test was conducted to determine whether respondents had interpretation problems and to ensure that the experimental scenarios were applicable to this group of subjects. This notwithstanding, exercise intensity may not have been adequately described as it could be difficult for a layperson to comprehend what high, medium and low exercise intensity could be without specific examples. The questionnaire was not validated prior to testing on study participants. Personality traits (such as openness, extraversion etc.) have not been explicitly measured although other research findings support the notion that such individual differences may contribute to preference for exercise and acceptance to engage in some type of physical activity [45, 46]. The sample size was not sufficient to conduct a stratified subgroup analysis. The results were however stable across the investigated stratified subgroup analysis that was performed. The sample population was random and dependent on the survey objective, allowing for generalization of the results to the population in question. There may however be differences between the study sample and the population in question that were not captured by the background variables. Finally, the two alternatives in this ‘forced-choice’ question format required individuals to pick an exercise when in reality they might not have chosen either. An opt-out option was not included because the evidence recommends that individuals with LBP should stay active. Further questions that asked participants to indicate their level of certainty about their choices would perhaps have clarified the extent of the bias caused by the forced choice of an exercise option.

Future research should examine the incentives for employers to implement and promote worker health. Since incentives are found to influence participation in exercise, evaluating the affordability of incentive use and their ability to encourage sustained physical activity among working adults should be examined in model programs. Further, in order to improve adherence to exercise among individuals with non-specific LBP, the role of relevant personality traits in influencing the preference for exercise needs to be further examined to understand the preference for exercise intensity [46], as well as other exercise attributes.

## Conclusions

When choosing exercise to prevent future episodes of non-specific LBP, the way individuals value the different components of exercise varies from one person to another. Intensity (high) and frequency (once to twice per week) of exercise and type of financial incentive (exercise during working hours) play an important role in individuals’ decision-making. These findings may be useful for the planning and development of exercise provision and promotion strategies for the secondary prevention of non-specific LBP.

## Supporting information

S1 File Choice task questions in English. (DOCX)

S2 File Choice task questions in Swedish. (DOCX)

S1 Tables Complementary sub-group analysis. (DOCX)
