# Supplementary material for: Individual preferences for physical exercise as secondary prevention for non-specific low back pain: A discrete choice experiment
Source: PLoS One. 2017 Dec 15;12(12):e0187709. doi: 10.1371/journal.pone.0187709 (PMC5731740; doi:10.1371/journal.pone.0187709)
Supplement: S1 File — (DOCX) [file pone.0187709.s001.docx]

**CHOICE TASK: EXERCISE PREFERENCES** **FOR LOW BACK PAIN**

**Instructions:**

Low back pain is a very common problem for many working people. Exercises has been proven to be effective in preventing recurrent back pain, so it is important to find out what type of exercise people prefer.

In the following questionnaire, you will be presented with two different options involving different exercises. For each of the following choices, please choose the ONE option you prefer, either option A or option B. Please tick only ONE box for each choice. Please read the questions carefully before answering all the questions. There is no right or wrong answer, but your personal opinions are important.

**Description of exercise attributes**

| **Attributes** | **Attribute levels** | **Description** |  |  |
| --- | --- | --- | --- | --- |
| Type of exercise | Strength training  Cardiovascular training  Mindfulness-based training | The kind of exercise that is performed (e.g. running, stretching, yoga). | |  |
| Design | Individual with supervision  Individual without supervision  Group with supervision  Group without supervision | Decision to exercise individually or in groups, and exercise that is supervised or not supervised. | |  |
| Intensity | Low  Medium  High | The degree of effort required to perform the physical exercise. | |  |
| Frequency | Once a week  Two times per week  Three times per week | The number of times per week the exercise is performed. | |  |
| Proximity | 10 minutes  20 minutes  30 minutes | Travel time to the location (e.g. gym) where you can exercise. regularly. | |  |
| Incentives | None  Discount coupon for sports goods  Wellness subsidies  Exercise in working hours (1h per week) week) | Describe incentives to receive from an employer as motivation for exercising. | | |

**Choice 1**

|  | Alternative **A** | Alternative **B** |
| --- | --- | --- |
| Type of exercise | Strength training | Strength training |
| Design | Individual without supervision | Individual without supervision |
| Intensity | Medium | Medium |
| Frequency | Two times per week | E Once a week |
| Proximity | 10 minutes | 10 minutes |
| Incentives | Exercise in working hours (1h per week) | Exercise in working hours (1h per week) |
| Which exercise option do you prefer? | **[ ]** | **[ ]** |

(Please check only one box)

**Choice 2**

|  | Alternative **A** | Alternative **B** |
| --- | --- | --- |
| Type of exercise | Mindfulness-based training | Strength training |
| Design | Group without supervision | Individual with supervision |
| Intensity | Low | High |
| Frequency | Once a week | Two times per week |
| Proximity | 10 minutes | 30 minutes |
| Incentives | Exercise in working hours (1h per week) | Wellness subsidies |
| Which exercise option do you prefer? | **[ ]** | **[ ]** |

(Please check only one box)

**Choice 3**

|  | Alternative **A** | Alternative **B** |
| --- | --- | --- |
| Type of exercise | Cardiovascular training | Strength training |
| Design | Group without supervision | Individual without supervision |
| Intensity | Low | Medium |
| Frequency | Once a week | Two times per week |
| Proximity | 10 minutes | 20 minutes |
| Incentives | None | Wellness subsidies |
| Which exercise option do you prefer? | **[ ]** | **[ ]** |

(Please check only one box)

**Choice 4**

|  | Alternative **A** | Alternative **B** |
| --- | --- | --- |
| Type of exercise | Mindfulness-based training | Cardiovascular training |
| Design | Group with supervision | Individual without supervision |
| Intensity | Medium | High |
| Frequency | Two times per week | Two times per week |
| Proximity | 30 minutes | 20 minutes |
| Incentives | None | Discount coupon for sports goods |
| Which exercise option do you prefer? | **[ ]** | **[ ]** |

(Please check only one box)

**Choice 5**

|  | Alternative **A** | Alternative **B** |
| --- | --- | --- |
| Type of exercise | Cardiovascular training | Strength training |
| Design | Individual without supervision | Group with supervision |
| Intensity | Medium | Low |
| Frequency | Once a week | Three times per week |
| Proximity | 20 minutes | 20 minutes |
| Incentives | Exercise in working hours (1h per week) | Wellness subsidies |
| Which exercise option do you prefer? | **[ ]** | **[ ]** |

(Please check only one box)

**Choice 6**

|  | Alternative **A** | Alternative **B** |
| --- | --- | --- |
| Type of exercise | Mindfulness-based training | Mindfulness-based training |
| Design | Individual without supervision | Group with supervision |
| Intensity | High | Low |
| Frequency | Two times per week | Once a week |
| Proximity | 10 minutes | 20 minutes |
| Incentives | Discount coupon for sports goods | Wellness subsidies |
| Which exercise option do you prefer? | **[ ]** | **[ ]** |

(Please check only one box)

**Choice 7**

|  | Alternative **A** | Alternative **B** |
| --- | --- | --- |
| Type of exercise | Strength training | Cardiovascular training |
| Design | Group with supervision | Group with supervision |
| Intensity | Low | High |
| Frequency | Once a week | Once a week |
| Proximity | 20 minutes | 30 minutes |
| Incentives | Wellness subsidies | Discount coupon for sports goods |
| Which exercise option do you prefer? | **[ ]** | **[ ]** |

(Please check only one box)

**Choice 8**

|  | Alternative **A** | Alternative **B** |
| --- | --- | --- |
| Type of exercise | Mindfulness-based training | Cardiovascular training |
| Design | Individual with supervision | Individual without supervision |
| Intensity | Low | Medium |
| Frequency | Three times per week | Once a week |
| Proximity | 20 minutes | 10 minutes |
| Incentives | Wellness subsidies | Exercise in working hours (1h per week) |
| Which exercise option do you prefer? | **[ ]** | **[ ]** |

(Please check only one box)

**Choice 9**

|  | Alternative **A** | Alternative **B** |
| --- | --- | --- |
| Type of exercise | Mindfulness-based traning | Strength training |
| Design | Group without supervision | Individual with supervision |
| Intensity | High | High |
| Frequency | Once a week | Two times per week |
| Proximity | 10 minutes | 30 minutes |
| Incentives | None | None |
| Which exercise option do you prefer? | **[ ]** | **[ ]** |

(Please check only one box)

**Choice 10**

|  | Alternative **A** | Alternative **B** |
| --- | --- | --- |
| Type of exercise | Cardiovascular training | Cardiovascular training |
| Design | Individual with supervision | Group with supervision |
| Intensity | Low | High |
| Frequency | Two times per week | Three times per week |
| Proximity | 10 minutes | 30 minutes |
| Incentives | Wellness subsidies | Discount coupon for sports goods |
| Which exercise option do you prefer? | **[ ]** | **[ ]** |

(Please check only one box)

**Avslutande frågor**

1. In the above questions you were asked to choose between different exercise options you preferred. Are there any other factors or characteristics, except those you have taken into consideration, which you consider important to choose to engage in exercise?

……………………………………………………………………………………………………………………………………………………………………………………………………………………………………………………………………………………………………………………………………………………………………………………………………………………………………………………………………………………

2. If you have any further comments on the answer options or questionnaire, feel free to write them here.

……………………………………………………………………………………………………………………

………………………………………………………………………………………………………………………………………………………………………………………………………………………………………………………………………………………………………………………………………………………………
