# Supplementary material for: Individual preferences for physical exercise as secondary prevention for non-specific low back pain: A discrete choice experiment
Source: PLoS One. 2017 Dec 15;12(12):e0187709. doi: 10.1371/journal.pone.0187709 (PMC5731740; doi:10.1371/journal.pone.0187709)
Supplement: S2 File — (DOCX) [file pone.0187709.s002.docx]

**CHOICE TASK**

**Frågor om val av träningsform**

Ryggsbesvär är ett mycket vanligt problem för många människor. Träning har vetenskapligt visat sig vara effektivt för att förebygga återkommande ryggsbesvär, därför är det viktigt att ta reda på vilken typ av träning som människor föredrar.

Nedan ser du en uppställning över vilka träningsprogram som används i enkäten. Läs noga igenom tabellen innan du besvarar frågorna på nästa sida. Du kommer därefter att få ett antal olika träningsbeskrivningar där du skall välja vilket alternativ som passar dig. Du kan gå tillbaka och titta på dessa förklaringar av träningsprogram när du vill.

Träningsprogrammen beskrivs genom följande egenskaper

| **Egenskaper** | **Vad som ingår i egenskaperna** | **Beskrivning av egenskaper** |
| --- | --- | --- |
| Typ av träning | Styrketräning  Konditionsträning  Mindfulness-baserad träning | Den form av träning som ska genomföras. Exempel på typ av träning; löpning, styrka, yoga, dans och liknande. |
| Träningsutformning | Individuellt med instruktör  Individuellt utan instruktör  Grupp med ledare  Grupp utan ledare | Beslut att träna ensam eller i grupp, samt en träning som är handledd eller inte. |
| Intensitet | Låg  Medel  Hög | Graden av ansträngning som krävs för att utföra träningen eller den fysiska aktiviteten. |
| Frekvens | En gång i veckan  Två gånger i veckan  Tre gånger i veckan | Hur många gånger träningen kan utföras per vecka. |
| Närheten till träningsplats | 10 minuter  20 minuter  30 minuter | Närhet till platsen (t ex. gym, simhall, lokaler) där du kan utföra träningen regelbundet. |
| Incitament till träning | Ingen ersättning  Rabattkupong till sportaffär  Friskvårdsbidrag  Träning på arbetstid (1h per vecka) | Beskriver vilken ersättning du skulle föredra från arbetsgivare som uppmuntran/stöd till din träning. |

Nedan följer tio olika alternativ av träningsprogram. Läs noga igenom dessa och svara sedan på de efterföljande frågorna. För varje val kryssar du endast det alternativ du föredrar, **antingen** alternativ A **eller** alternativ B.

Kombinationerna av egenskaper i valen nedanför kan tyckas ovanliga och kan vara svåra att svara på, men välj ändå ett alternativ för varje fråga, A eller B. Det finns inget rätt eller fel svar, utan det är dina personliga åsikter som är viktiga.

**Val 1**

|  | Alternativ **A** | Alternativ **B** |
| --- | --- | --- |
| Typ av träning | Styrketräning | Styrketräning |
| Träningsutformning | Individuellt utan instruktör | Individuellt utan instruktör |
| Intensitet | Medel | Medel |
| Frekvens | Två gånger i veckan | En gång i veckan |
| Närheten till träningsplats | 10 minuter | 10 minuter |
| Incitament till träning | Träning på arbetstid | Träning på arbetstid |
| Vilket träningsprogram skulle du föredra? | **[ ]** | **[ ]** |

(Kryssa endast i en ruta)

**Val 2**

|  | Alternativ **A** | Alternativ **B** |
| --- | --- | --- |
| Typ av träning | Mindfulness-baserad träning | Styrketräning |
| Träningsutformning | Grupp utan ledare | Individuellt med instruktör |
| Intensitet | Låg | Hög |
| Frekvens | En gång i veckan | Två gånger i veckan |
| Närheten till träningsplats | 10 minuter | 30 minuter |
| Incitament till träning | Träning på arbetstid | Friskvårdsbidrag |
| Vilket träningsprogram skulle du föredra? | **[ ]** | **[ ]** |

(Kryssa endast i en ruta)

**Val 3**

|  | Alternativ **A** | Alternativ **B** |
| --- | --- | --- |
| Typ av träning | Konditionsträning | Styrketräning |
| Träningsutformning | Grupp utan ledare | Individuellt utan instruktör |
| Intensitet | Låg | Medel |
| Frekvens | En gång i veckan | Två gånger i veckan |
| Närheten till träningsplats | 10 minuter | 20 minuter |
| Incitament till träning | Ingen ersättning | Friskvårdsbidrag |
| Vilket träningsprogram skulle du föredra? | **[ ]** | **[ ]** |

(Kryssa endast i en ruta)

**Val 4**

|  | Alternativ **A** | Alternativ **B** |
| --- | --- | --- |
| Typ av träning | Mindfulness-baserad träning | Konditionsträning |
| Träningsutformning | Grupp med ledare | Individuellt utan instruktör |
| Intensitet | Medel | Hög |
| Frekvens | Två gånger i veckan | Två gånger i veckan |
| Närheten till träningsplats | 30 minuter | 20 minuter |
| Incitament till träning | Ingen ersättning | Rabattkupong till sportaffär |
| Vilket träningsprogram skulle du föredra? | **[ ]** | **[ ]** |

(Kryssa endast i en ruta)

**Val 5**

|  | Alternativ **A** | Alternativ **B** |
| --- | --- | --- |
| Typ av träning | Konditionsträning | Styrketräning |
| Träningsutformning | Individuellt utan instruktör | Grupp med ledare |
| Intensitet | Medel | Låg |
| Frekvens | En gång i veckan | Tre gånger i veckan |
| Närheten till träningsplats | 20 minuter | 20 minuter |
| Incitament till träning | Träning på arbetstid | Friskvårdsbidrag |
| Vilket träningsprogram skulle du föredra? | **[ ]** | **[ ]** |

(Kryssa endast i en ruta)

**Val 6**

|  | Alternativ **A** | Alternativ **B** |
| --- | --- | --- |
| Typ av träning | Mindfulness-baserad träning | Mindfulness-baserad träning |
| Träningsutformning | Individuellt utan instruktör | Grupp med ledare |
| Intensitet | Hög | Låg |
| Frekvens | Två gånger i veckan | En gång i veckan |
| Närheten till träningsplats | 10 minuter | 20 minuter |
| Incitament till träning | Rabattkupong till sportaffär | Friskvårdsbidrag |
| Vilket träningsprogram skulle du föredra? | **[ ]** | **[ ]** |

(Kryssa endast i en ruta)

**Val 7**

|  | Alternativ **A** | Alternativ **B** |
| --- | --- | --- |
| Typ av träning | Styrketräning | Konditionsträning |
| Träningsutformning | Grupp med ledare | Grupp med ledare |
| Intensitet | Låg | Hög |
| Frekvens | En gång i veckan | En gång i veckan |
| Närheten till träningsplats | 20 minuter | 30 minuter |
| Incitament till träning | Friskvårdsbidrag | Rabattkupong till sportaffär |
| Vilket träningsprogram skulle du föredra? | **[ ]** | **[ ]** |

(Kryssa endast i en ruta)

**Val 8**

|  | Alternativ **A** | Alternativ **B** |
| --- | --- | --- |
| Typ av träning | Mindfulness-baserad träning | Konditionsträning |
| Träningsutformning | Individuellt med instruktör | Individuellt utan instruktör |
| Intensitet | Låg | Medel |
| Frekvens | Tre gånger i veckan | En gång i veckan |
| Närheten till träningsplats | 20 minuter | 10 minuter |
| Incitament till träning | Friskvårdsbidrag | Träning på arbetstid |
| Vilket träningsprogram skulle du föredra? | **[ ]** | **[ ]** |

(Kryssa endast i en ruta)

**Val 9**

|  | Alternativ **A** | Alternativ **B** |
| --- | --- | --- |
| Typ av träning | Mindfulness-baserad träning | Styrketräning |
| Träningsutformning | Grupp utan ledare | Individuellt med instruktör |
| Intensitet | Hög | Hög |
| Frekvens | En gång i veckan | Två gånger i veckan |
| Närheten till träningsplats | 10 minuter | 30 minuter |
| Incitament till träning | Ingen ersättning | Ingen ersättning |
| Vilket träningsprogram skulle du föredra? | **[ ]** | **[ ]** |

(Kryssa endast i en ruta)

**Val 10**

|  | Alternativ **A** | Alternativ **B** |
| --- | --- | --- |
| Typ av träning | Konditionsträning | Konditionsträning |
| Träningsutformning | Individuellt med instruktör | Grupp med ledare |
| Intensitet | Låg | Hög |
| Frekvens | Två gånger i veckan | Tre gånger i veckan |
| Närheten till träningsplats | 10 minuter | 30 minuter |
| Incitament till träning | Friskvårdsbidrag | Rabattkupong till sportaffär |
| Vilket träningsprogram skulle du föredra? | **[ ]** | **[ ]** |

(Kryssa endast i en ruta)

**Avslutande frågor**

1. I frågorna ovan fick du välja mellan olika träningsalternativ. Finns det några andra faktorer eller egenskaper, förutom de som du fått ta ställning till, som du anser vara viktiga för att välja att träna?

……………………………………………………………………………………………………………………………………………………………………………………………………………………………………………………………………………………………………………………………………………………………………………………………………………………………………………………………………………………

2. Om du har några ytterligare kommentarer till svarsalternativen eller enkäten så kan du gärna skriva dem här.

……………………………………………………………………………………………………………………

………………………………………………………………………………………………………………………………………………………………………………………………………………………………………………………………………………………………………………………………………………………………
