# Supplementary material for: Individual preferences for physical exercise as secondary prevention for non-specific low back pain: A discrete choice experiment
Source: PLoS One. 2017 Dec 15;12(12):e0187709. doi: 10.1371/journal.pone.0187709 (PMC5731740; doi:10.1371/journal.pone.0187709)
Supplement: S1 Tables — (DOCX) [file pone.0187709.s003.docx]

# SUB-GROUP ANALYSIS OF EXERCISE PREFERENCES BASED ON SUBJECTS’ BACKGROUND CHARACTERISTICS

**Sub-group analysis: Exercise preferences influenced by individual characteristics, gender**

| **Attribute levels** | **Coefficient [Gender (0) = Male], n = 45** | **Coefficient [Gender (1) = Female], n = 67** | **Difference [Coefficients]** | **p-value** | **[95% Conf. Interval]** |
| --- | --- | --- | --- | --- | --- |
| Cardiovascular training | 1.14 | 1.09 | 0.04 | 0.913 | -0.69; 0.78 |
| Mindfulness-based training | 0.07 | 0.256 | -0.19 | 0.565 | -0.84; 0.46 |
| Strength training | Set to 0 | Set to 0 |  |  |  |
| Individual with supervision | 0.22 | 0.59 | -0.37 | 0.574 | -1.64; 0.91 |
| Individual without supervision | 1.50 | 0.56 | 0.94 | 0.408 | -1.29; 3.16 |
| Group with supervision | 1.44 | 1.21 | 0.234 | 0.683 | -0.91; 1.39 |
| Group without supervision | Set to 0 | Set to 0 |  |  |  |
| Low intensity | 0.54 | 0.11 | 0.43 | 0.653 | -1.45; 2.31 |
| High intensity | 2.21 | 1.11 | 1.10 | 0.173 | -0.48; 2.69 |
| Medium intensity | Set to 0 | Set to 0 |  |  |  |
| Once a week (Frequency) | 0.49 | 0.98 | -0.48 | 0.194 | -1.21; 0.25 |
| 2 times/ week (Frequency) | 1.77 | 1.61 | 0.16 | 0.694 | -0.65; 0.98 |
| 3 times/ week | Set to 0 | Set to 0 |  |  |  |
| Proximity (10 minutes) | 0.12 | 0.46 | -0.33 | 0.704 | -2.04; 1.38 |
| Proximity (20 mintues) | 0.22 | 0.70 | -0.48 | 0.623 | -2.40; 1.44 |
| Proximity (30 mintues) | Set to 0 | Set to 0 |  |  |  |
| None (Incentives) | 2.37 | 1.71 | 0.66 | 0.474 | -1.15; 2.48 |
| Wellness subsidies | 3.59 | 2.26 | 1.33 | 0.203 | -0.72; 3.38 |
| Exercise at work | 3.37 | 2.46 | 0.92 | 0.342 | -0.97; 2.81 |
| Discount coupon for sports goods | Set to 0 | Set to 0 |  |  |  |

Number of observations (Male) = 900; Number of observations (Female) = 1340; Set to 0 (Zero) = Reference category

| **Sub-group analysis: Exercise preferences influenced by individual characteristics, physical activity level** | | | | | |
| --- | --- | --- | --- | --- | --- |
| **Attribute levels** | **Coefficient [Inactive], n = 37** | **Coefficient [Active], n = 74** | **Difference [Coefficients]** | **p-value** | **[95% Conf. Interval]** |
| Cardiovascular training | 1.03 | 1.13 | -0.09 | 0.815 | -0.87; 0.68 |
| Mindfulness-based training | 0.09 | 0.19 | -0.11 | 0.745 | -0.80; 0.57 |
| Strength training | Set to 0 | Set to 0 |  |  |  |
| Individual with supervision | 1.38 | 0.12 | 1.25 | 0.062 | -0.06; 2.57 |
| Individual without supervision | 0.67 | 0.91 | -0.24 | 0.845 | -2.60; 2.13 |
| Group with supervision | 1.68 | 1.15 | 0.53 | 0.372 | -0.63; 1.68 |
| Group without supervision | Set to 0 | Set to 0 |  |  |  |
| Low intensity | 0.28 | 0.23 | 0.05 | 0.958 | -1.93; 2.03 |
| High intensity | 1.11 | 1.66 | -0.55 | 0.512 | -2.20; 1.09 |
| Medium intensity | Set to 0 | Set to 0 |  |  |  |
| Once a week (Frequency) | 1.54 | 0.51 | 1.03 | **0.015** | 0.20; 1.86 |
| 2 times/ week (Frequency) | 2.54 | 1.39 | 1.15 | **0.012** | 0.25; 2.06 |
| 3 times/ week | Set to 0 | Set to 0 |  |  |  |
| Proximity (10 minutes) | 0.97 | 0.26 | 0.71 | 0.441 | -1.10; 2.53 |
| Proximity (20 mintues) | 1.45 | 0.32 | 1.13 | 0.269 | -0.87; 3.13 |
| Proximity (30 mintues) | Set to 0 | Set to 0 |  |  |  |
| None (Incentives) | 1.79 | 1.97 | -0.18 | 0.853 | -2.09; 1.73 |
| Wellness subsidies | 2.15 | 2.90 | -0.75 | 0.484 | -2.86; 1.35 |
| Exercise at work | 2.73 | 2.76 | -0.03 | 0.973 | -1.99; 1.92 |
| Discount coupon for sports goods | Set to 0 | Set to 0 |  |  |  |
| Number of observations (inactive) = 740; Number of observations (active) = 1480; Set to 0 (Zero) = Reference category | | | | | |

**Sub-group analysis: Exercise preferences influenced by individual characteristics, number of children at home**

| **Attribute levels** | **Coefficient [Children = 0], n = 48** | **Coefficient [Children ≥ 1], n = 58** | **Difference [Coefficients]** | **p-value** | **[95% Conf. Interval]** |
| --- | --- | --- | --- | --- | --- |
| Cardiovascular training | 0.84 | 1.49 | -0.65 | 0.091 | -1.40; 0.10 |
| Mindfulness-based training | 0.12 | 0.27 | -0.15 | 0.658 | -0.81; 0.51 |
| Strength training | Set to 0 | Set to 0 |  |  |  |
| Individual with supervision | 0.73 | 0.03 | 0.70 | 0.279 | -0.57; 1.97 |
| Individual without supervision | -0.73 | 2.37 | -3.10 | **0.007** | -5.37; -0.84 |
| Group with supervision | 1.19 | 1.49 | -0.31 | 0.599 | -1.44; 0.83 |
| Group without supervision | Set to 0 | Set to 0 |  |  |  |
| Low intensity | -1.12 | 1.37 | -2.49 | **0.009** | -4.39; -0.59 |
| High intensity | 0.59 | 2.56 | -1.97 | **0.016** | -3.58; -0.36 |
| Medium intensity | Set to 0 | Set to 0 |  |  |  |
| Once a week (Frequency) | 0.88 | 0.76 | 0.12 | 0.755 | -0.63; 0.87 |
| 2 times/ week (Frequency) | 1.94 | 1.58 | 0.36 | 0.393 | -0.47; 1.19 |
| 3 times/ week | Set to 0 | Set to 0 |  |  |  |
| Proximity (10 minutes) | 1.36 | -0.46 | 1.83 | **0.035** | 0.13; 3.53 |
| Proximity (20 mintues) | 1.84 | -0.55 | 2.39 | **0.014** | 0.48; 4.31 |
| Proximity (30 mintues) | Set to 0 | Set to 0 |  |  |  |
| None (Incentives) | 1.05 | 2.97 | -1.91 | **0.042** | -3.76; -0.07 |
| Wellness subsidies | 2.13 | 3.74 | -1.61 | 0.126 | -3.68; 0.45 |
| Exercise at work | 2.14 | 3.73 | -1.59 | 0.103 | -3.51; 0.32 |
| Discount coupon for sports goods | Set to 0 | Set to 0 |  |  |  |

Number of observations = 960 for those with no child; Number of observations = 1160 for those with more than one child; Set to 0 (Zero) = Reference category

**Sub-group analysis: Exercise preferences influenced by individual characteristics, completed level of education**

| **Attribute levels** | **Coefficient [Basic education = 0]** | **Coefficient [Higher education = 1]** | **[Coefficients]**  **Difference** | **p-value** | **[95% Conf. Interval]** |
| --- | --- | --- | --- | --- | --- |
| Cardiovascular training | 0.96 | 1.31 | -0.36 | 0.333 | -1.08; 0.37 |
| Mindfulness-based training | 0.08 | 0.31 | -0.22 | 0.489 | -0.86; 0.41 |
| Strength training | Set to 0 | Set to 0 |  |  |  |
| Individual with supervision | 0.81 | 0.02 | 0.78 | 0.206 | -0.43; 1.99 |
| Individual without supervision | 0.58 | 1.36 | -0.77 | 0.486 | -2.95; 1.40 |
| Group with supervision | 1.36 | 1.23 | 0.13 | 0.811 | -0.95; 1.22 |
| Group without supervision | Set to 0 | Set to 0 |  |  |  |
| Low intensity | 0.03 | 0.63 | -0.60 | 0.517 | -2.43; 1.22 |
| High intensity | 1.19 | 1.94 | -0.75 | 0.339 | -2.29; 0.79 |
| Medium intensity | Set to 0 | Set to 0 |  |  |  |
| Once a week (Frequency) | 0.97 | 0.57 | 0.40 | 0.271 | -0.32; 1.12 |
| 2 times/ week (Frequency) | 1.87 | 1.45 | 0.42 | 0.302 | -0.38; 1.22 |
| 3 times/ week | Set to 0 | Set to 0 |  |  |  |
| Proximity (10 minutes) | 0.82 | -0.23 | 1.06 | 0.205 | -0.58; 2.69 |
| Proximity (20 mintues) | 1.03 | -0.089 | 1.12 | 0.232 | -0.72; 2.97 |
| Proximity (30 mintues) | Set to 0 | Set to 0 |  |  |  |
| None (Incentives) | 1.63 | 2.37 | -0.74 | 0.414 | -2.51; 1.03 |
| Wellness subsidies | 2.33 | 3.24 | -0.91 | 0.370 | -2.89; 1.08 |
| Exercise at work | 2.42 | 3.27 | -0.86 | 0.362 | -2.69; 0.98 |
| Discount coupon for sports goods | Set to 0 | Set to 0 |  |  |  |

Number of observations = 1280 with basic education; Number of observations with higher education = 960; Set to 0 (Zero) = Reference category

**Sub-group analysis: Exercise preferences influenced by individual characteristics, neck and back pain**

| **Attribute levels** | **Coefficient [Neck and back pain (0) = Yes]** | **Coefficient [Neck and back pain (1) = No]** | | **Difference**  **[Coefficients]** | | | **p-value** | **[95% Conf. Interval]** | |  |
| --- | --- | --- | --- | --- | --- | --- | --- | --- | --- | --- |
| Cardiovascular training | 1.03 | | 1.31 | | -0.28 | 0.505 | | | 0.53; -0.67 | |
| Mindfulness-based training | 0.14 | | 0.29 | | -0.15 | 0.683 | | | 0.57; -0.41 | |
| Strength training | Set to 0 | | Set to 0 | |  |  | | |  | |
| Individual with supervision | 0.55 | | 0.22 | | 0.33 | 0.636 | | | 1.68; -0.47 | |
| Individual without supervision | 1.02 | | 0.67 | | 0.35 | 0.780 | | | 2.79; -0.28 | |
| Group with supervision | 1.42 | | 0.94 | | 0.48 | 0.443 | | | 1.69; -0.77 | |
| Group without supervision | Set to 0 | | Set to 0 | |  |  | | |  | |
| Low intensity | 0.35 | | 0.14 | | 0.21 | 0.842 | | | 2.25; -0.19 | |
| High intensity | 1.53 | | 1.46 | | 0.08 | 0.931 | | | 1.81; -0.09 | |
| Medium intensity | Set to 0 | | Set to 0 | |  |  | | |  | |
| Once a week (Frequency) | 0.82 | | 0.71 | | 0.11 | 0.794 | | | 0.91; -0.26 | |
| 2 times/ week (Frequency) | 1.71 | | 1.58 | | 0.13 | 0.769 | | | 1.02; -0.29 | |
| 3 times/ week | Set to 0 | | Set to 0 | |  |  | | |  | |
| Proximity (10 minutes) | 0.46 | | 0.03 | | 0.42 | 0.651 | | | 2.25; -0.45 | |
| Proximity (20 mintues) | 0.62 | | 0.29 | | 0.33 | 0.756 | | | 2.39; -0.31 | |
| Proximity (30 mintues) | Set to 0 | | Set to 0 | |  |  | | |  | |
| None (Incentives) | 2.08 | | 1.61 | | 0.47 | 0.646 | | | 2.46; -0.46 | |
| Wellness subsidies | 2.81 | | 2.45 | | 0.35 | 0.756 | | | 2.59; -0.31 | |
| Exercise at work | 2.83 | | 2.64 | | 0.19 | 0.859 | | | 2.26; -0.18 | |
| Discount coupon for sports goods | Set to 0 | | Set to 0 | |  |  | | |  | |

Number of observations with neck and back pain = 1660; Number of observations = with no neck and back pain 580; Set to 0 (Zero) = Reference category
